# Adaptive and Behavioral Phenotype in Pediatric 22q11.2 Deletion Syndrome: Characterizing a High-Risk Neurogenetic Copy Number Variant

**DOI:** 10.3390/genes17020120

**Published:** 2026-01-24

**Authors:** Larissa Salustiano Evangelista Pimenta, Claudia Berlim de Mello, Guilherme V. Polanczyk, Leslie Domenici Kulikowski, Maria Isabel Melaragno, Chong Ae Kim

**Affiliations:** 1 Genetics Unit, Instituto da Criança, Faculdade de Medicina FMUSP, Universidade de São Paulo, São Paulo 05403-000, Brazil; 2 Departament of Psychobiology, Universidade Federal de São Paulo, São Paulo 04024-002, Brazil; 3 Department & Institute of Psychiatry of Medicine, Faculdade de Medicina FMUSP, Universidade de São Paulo, São Paulo 05401-000, Brazil; 4 Cytogenomics Laboratory, Departament of Pathology, Faculdade de Medicina FMUSP, Universidade de São Paulo, São Paulo 01246-903, Brazil; 5 Genetics Division, Department of Morphology and Genetics, Universidade Federal de São Paulo, São Paulo 04023-062, Brazil

**Keywords:** 22q11.2 deletion syndrome, copy number variants, adaptive functioning, behavior problems, neurodevelopmental disorders genotype-phenotype correlations

## Abstract

22q11.2 deletion syndrome (22q11.2DS) is the most common recurrent microdeletion in humans and a prototypical high-risk neurogenetic copy number variant (CNV) associated with a broad spectrum of neurodevelopmental and psychiatric disorders, including intellectual disability (ID), autism spectrum disorder (ASD), attention-deficit/hyperactivity disorder (ADHD), anxiety, and psychotic symptoms. This hemizygous deletion encompasses multiple genes involved in brain development and neural circuit function, contributing to marked phenotypic variability and multisystem involvement. In pediatric populations, deficits in adaptive functioning are frequently reported and may occur independently of global intellectual impairment, reflecting broader behavioral vulnerabilities within this genetic risk architecture. **Background/Objectives:** This study aimed to characterize the sociodemographic, clinical, and intellectual profiles of children and adolescents with 22q11.2DS and to examine adaptive functioning and its associations with behavioral difficulties. **Methods:** Thirty-four patients aged 1–17 years with a confirmed molecular diagnosis of 22q11.2DS were assessed. Standardized instruments were used to evaluate cognitive performance, adaptive functioning, and behavioral outcomes. **Results:** Intellectual disability was highly prevalent, with most participants showing combined cognitive and adaptive impairments. Adaptive functioning was compromised across domains, with relatively higher socialization scores compared to other areas, such as daily living skills. Multivariate analyses indicated associations between sociodemographic factors and behavioral difficulties, as well as between social problems and lower global adaptive functioning. **Conclusions:** Together, these findings contribute to the characterization of the adaptive and behavioral phenotype associated with a high-risk neurogenetic CNV and highlight the relevance of adaptive functioning as a key outcome for early evaluation and intervention in pediatric 22q11.2DS.

## 1. Introduction

The 22q11.2 deletion syndrome (22q11.2DS) results from a submicroscopic deletion on the long arm of chromosome 22 (region 22q11.2; OMIM #192430 and #188400). It represents one of the most common pathogenic copy number variants (CNVs) in humans and a prototypical high-risk neurogenetic model. Recognized as the most prevalent microdeletion syndrome in humans, it involves the haploinsufficiency of approximately 50–60 genes and presents with a wide spectrum of clinical, neurodevelopmental, and neurobehavioral manifestations [1,2,3].

Regardless of the extent of the deletion, the clinical phenotype of 22q11.2DS exhibits wide variability. The most frequent manifestations include congenital cardiac and palatal malformations, immunological and autoimmune dysfunctions, and endocrine, genitourinary, gastrointestinal, and neurological abnormalities. Delays in neuropsychomotor development are common, along with cognitive deficits and a broad range of neuropsychiatric disorders, highlighting the multisystem and pleiotropic impact of this genetic lesion [2,3,4].

A major area of research interest in 22q11.2DS concerns the high prevalence of psychiatric disorders, which occur at markedly higher rates and with greater comorbidity compared to the general population [5,6]. In pediatric patients with 22q11.2DS, elevated rates of anxiety symptoms are frequently observed. With increasing age, these symptoms often evolve into more complex clinical presentations, including generalized anxiety disorder, specific phobias, and social anxiety disorder—conditions that are sometimes associated with the emergence of psychotic-like experiences [6,7,8].

Neurodevelopmental disorders are also widely documented in this population. Among the most prevalent conditions in childhood are attention-deficit/hyperactivity disorder (ADHD) and autism spectrum disorder (ASD), both reported at substantially higher rates than in typically developing peers. Disruptive behavior disorders, although also described, occur less frequently and tend to decline with increasing age [9].

Intellectual performance in 22q11.2 deletion syndrome (22q11.2DS) is characterized by marked heterogeneity, with full-scale IQ (FSIQ) scores ranging from the average range to levels consistent with intellectual disability [10,11,12,13]. Borderline intellectual functioning predominates, accounting for more than half of reported cases [11,14,15], while intellectual disability is also frequently observed, affecting approximately 40% of individuals with the syndrome [11,16,17,18].

Deficits in adaptive functioning—understood as the set of conceptual, social, and practical skills required to meet everyday life demands [19]—are closely associated with the intellectual disability profile observed in 22q11.2DS. In this study, adaptive functioning is conceptualized as a functional outcome that reflects how cognitive abilities, behavioral characteristics, and environmental factors converge in everyday performance. Previous studies have shown that both full-scale IQ (FSIQ) and adaptive functioning scores typically fall approximately two standard deviations below population norms, indicating a global pattern of impaired functioning in this population [6].

In children and adolescents with 22q11.2DS, adaptive functioning shows wide interindividual variability and a consistent pattern of discrepancy, with adaptive skills generally falling below intellectual capacities. This gap highlights the divergence between cognitive potential and actual performance in daily contexts [20,21]. Analyses of specific adaptive functioning domains in 22q11.2DS have shown that scores related to socialization abilities are significantly higher than those observed in other domains, such as daily living skills, which involve autonomy in personal care and everyday activities [21]. The socialization domain was also significantly associated with full-scale IQ (FSIQ), suggesting that cognitive performance plays an important role in the adaptive trajectory of these individuals [21,22].

Adaptive functioning has also been linked to behavioral and emotional difficulties in children and adolescents with 22q11.2DS. Studies examining the relationship between emotional symptoms and adaptive functioning have found that anxiety symptoms are negatively associated with adaptive outcomes, contributing to more limited everyday performance [7,23]. Furthermore, depressive symptoms in adolescents have also been associated with poorer adaptive functioning [7]. Although these findings highlight important vulnerabilities during early developmental stages, most research on adaptive functioning in 22q11.2DS has focused on characterizing its features in adulthood [24].

Given that 22q11.2DS is both a strong genetic risk factor for neurodevelopmental and psychiatric disorders and a clinically heterogeneous condition, a better understanding of adaptive functioning in this population is essential for delineating its behavioral phenotype. Considering the complexity of the intellectual profile and the influence of adaptive functioning on behavioral outcomes in 22q11.2DS, it is assumed that the underlying genetic liability conferred by the 22q11.2 deletion interacts with environmental and contextual factors as potential modulators of these processes. Accordingly, in the present study, adaptive functioning is conceptualized primarily as the main functional outcome, through which genetic vulnerability and sociodemographic and behavioral factors are expressed in everyday functioning. This integrative perspective enables a broader understanding of the individual variability observed in the syndrome and guides the development of more effective and contextually grounded interventions throughout the developmental course.

## 2. Materials and Methods

### 2.1. Participants

The sample consisted of 34 patients (Table 1), aged between 1 and 17 years (M = 11.5), equally distributed by sex: 17 females and 17 males.

All participants were followed at the Medical Genetics Unit, Instituto da Criança Prof. Pedro de Alcântara, Hospital das Clínicas, Faculty of Medicine, University of São Paulo (USP), Brazil. Clinical diagnoses were established by a medical geneticist and confirmed through molecular testing using fluorescence in situ hybridization (FISH), multiplex ligation-dependent probe amplification (MLPA), or comparative genomic hybridization (CGH array).

Regarding the nature of the deletion, approximately 90% of participants carried the typical 3 Mb deletion, encompassing 28 to 60 genes described in the literature. Concerning inheritance patterns, only 12% of cases were of maternal origin.

Written informed consent, approved by the Research Ethics Committee, was obtained from the legal guardians and the participants.

### 2.2. Procedures

#### 2.2.1. Sociodemographic and Clinical Variables

Family socioeconomic status (SES) was classified according to the Brazilian Economic Classification Criteria (ABEP) using a self-report questionnaire that evaluates variables such as asset ownership and access to services, educational level of the primary caregiver, and gross monthly household income. The total score derived from these indicators was used to assign families to specific socioeconomic strata [25].

Information regarding additional demographic variables—such as type of school attended (public or private), access to specialized educational and healthcare services, and clinical findings—was obtained through medical record review and/or reported by the primary caregiver during a structured anamnesis interview conducted by the neuropsychologist who subsequently performed the assessments with the patients.

#### 2.2.2. Cognition, Intellectual Assessment, and Adaptive Functioning

To screen cognitive development, participants under 2 years of age were assessed using the Bayley Scales of Infant and Toddler Development, designed for children aged 1 to 42 months. This instrument primarily aims to identify developmental delays and provide guidance for targeted interventions. In the present study, only the cognitive development scale was administered [26].

To describe intellectual profiles, age-appropriate instruments standardized for the Brazilian population were employed. Children aged between 2 years and 11 months and 6 years were evaluated using the SON-R Nonverbal Intelligence Test, which primarily measures nonverbal domains of intelligence for this age range [27].

Participants older than 6 years were assessed using the Wechsler Scales validated for the Brazilian population: the Wechsler Abbreviated Scale of Intelligence (WASI) and the Wechsler Intelligence Scale for Children—Fourth Edition (WISC-IV) [28,29].

Finally, adaptive functioning was evaluated using the Vineland Adaptive Behavior Scales (Second and Third Editions), completed by the primary caregiver. The scale assesses adaptive functioning across multiple domains, including communication, daily living skills, socialization, and motor skills [30,31].

#### 2.2.3. Behavioral Indicators Assessment

To assess indicators of internalizing and externalizing behavioral problems in children and adolescents, the Child Behavior Checklist (CBCL 6–18) was administered. The CBCL is a standardized questionnaire designed to evaluate social competence and behavioral problems in individuals aged 6 to 18 years, based on information provided by the primary caregiver [32].

### 2.3. Data Analysis

Statistical analyses were conducted using IBM SPSS Statistics (version 25). Initially, descriptive statistics were performed to characterize the sample, including absolute and relative frequencies for categorical variables, as well as measures of central tendency and dispersion for continuous variables.

In the exploratory stage, univariate linear regression analyses (also referred to as bivariate) were carried out to investigate preliminary associations between each independent variable and the study’s main outcomes. For the selection of candidate variables for the multiple models, an inclusion criterion of *p* < 0.20 was adopted, as widely recommended in epidemiological and psychometric studies, in order to avoid the exclusion of potentially relevant predictors [33].

Subsequently, parsimonious multivariate models (Appendix A) were constructed, simultaneously considering the previously selected independent variables, while maintaining the distinction between sociodemographic and clinical variables. When four or more eligible independent variables were present, only the three with the greatest statistical significance were retained, to avoid model overfitting and to respect the reduced sample size.

To increase the robustness of the estimates given the limited sample size (*n* = 34), the bootstrapping procedure with 5000 resamples was applied. This technique allowed the calculation of bias-corrected confidence intervals and adjusted estimates for the regression coefficients, thereby increasing the reliability of the results even in small samples. The significance level adopted in all analyses was 5% (*p* < 0.05). Non-standardized regression coefficients (B) were reported, along with their respective 95% confidence intervals (95% CI) obtained through bootstrapping, as well as *p*-values. In the multiple models, the adjusted R^2^ and F-statistic were also presented as global indicators of model fit.

## 3. Results

In the sociodemographic characterization of participants (Table 1), most children (59%) were enrolled in public schools, with three attending specialized classes in institutions for special education. Two participants were not yet attending school due to their young age, while one was out of the educational system by family decision, owing to adaptation difficulties and accessibility barriers in the academic environment.

Regarding learning difficulties, all caregivers reported some form of academic challenge, including preschool learning delays, weaknesses in literacy acquisition, and specific difficulties in areas such as mathematics and reading comprehension. However, only 50% of participants had access to inclusive educational resources within their schools, such as resource classrooms, adapted materials, a support teacher, or a school life assistant. Notably, 35% of children at the expected literacy stage had not yet achieved reading and writing proficiency.

Concerning caregiver education, 27 out of 34 primary caregivers reported having completed either secondary or higher education.

According to the Brazilian Economic Classification Criteria, 64% of families were classified within socioeconomic strata B1, B2, C1, or C2, corresponding to average monthly household incomes ranging from R$ 2403.04 to R$ 12,683.34—broadly representative of Brazil’s middle-class population [25].

Clinical data were obtained exclusively from medical records. All patients exhibited phenotypic features consistent with 22q11.2 deletion syndrome, including long face, hypertelorism, high-arched palate, and bulbous nasal tip. Major malformations were identified in 35% of cases, whereas neurological abnormalities such as epilepsy, microcephaly, and encephalopathy were observed in 21%. Congenital heart defects were present in 71% of participants, most frequently tetralogy of Fallot, ventricular septal defect, and interruption of the aortic arch.

Perinatal complications were also common: gestational issues such as oligohydramnios, intrauterine growth restriction, and maternal hypertension were reported in 27% of pregnancies, and neonatal complications (cyanosis, tachypnea, hypocalcemia, or respiratory distress) in 50%. Severe clinical conditions in early childhood were documented in 85% of medical records, including recurrent infections, gastroesophageal reflux, and cardiorespiratory complications. The most frequent surgical procedures were adenoidectomy, herniorrhaphy, palatoplasty, tympanostomy with ventilation tube insertion, and corrective cardiac surgeries.

Additional information was obtained through an anamnesis interview conducted with the primary caregiver. Developmental delay was reported in 82% of cases, predominantly affecting language; notably, four participants older than 5 years had not yet developed oral speech.

Behavioral difficulties of varying intensity were described by all caregivers, including symptoms of anxiety, mood instability, immaturity, psychomotor agitation, stereotypies, socialization difficulties, and emotional regulation impairments. Only 32% of participants were receiving continuous psychopharmacological treatment (fluoxetine, risperidone, chlorpromazine, or clonazepam) and/or psychiatric follow-up.

Approximately 53% of patients were engaged in multidisciplinary care, receiving interventions such as psychological counseling, speech therapy, and other specialized services.

Regarding Full-Scale IQ (FSIQ) (Table 2), assessed using the Wechsler Intelligence Scales (WISC-IV and WASI), the sample mean indicated intellectual functioning within the deficit range (M = 69.3). The highest observed score (FSIQ = 105) fell within the average range of intelligence, whereas the lowest score (FSIQ = 45) was two standard deviations below the normative mean for the Brazilian population. In three cases, significant difficulties in task comprehension and execution rendered test administration infeasible.

Among participants under 6 years of age, cognitive development was assessed using the Bayley Scales of Infant and Toddler Development (Cognitive Domain) and the nonverbal SON-R Intelligence Test. Three of these children exhibited clear signs of cognitive delay and/or deficits in nonverbal intellectual abilities.

With respect to Global Adaptive Functioning (GAF), evaluated through the Vineland Adaptive Behavior Scales based on caregiver reports, the mean performance of the sample was within the borderline range (M = 70.4), a pattern also observed in the Socialization domain. Conversely, the Communication, Daily Living Skills, and Motor Skills domains showed scores approximately two standard deviations below the normative mean, consistent with deficit-level performance.

Behavioral assessment, conducted using the Child Behavior Checklist (CBCL) completed by the primary caregiver, revealed clinical indicators of internalizing problems, particularly within the Withdrawn/Depressed domain, characterized by social withdrawal and reduced engagement in group activities—features often associated with depressive mood symptoms.

Additionally, significant alterations were observed in the Thought Problems domain, characterized by disorganized or tangential speech, difficulties in thought coherence, and impairments in peer relationships.

To further explore the findings, multivariate linear regression models were constructed using Full-Scale IQ (FSIQ) and Global Adaptive Functioning (GAF) as outcome variables. Only independent variables with *p* values below 0.20 in the univariate analysis were included, in order to respect the small sample size (n = 34) and minimize model overfitting. In each model, the three predictors showing the strongest associations with the respective outcomes were retained.

Subsequently, the multivariate models were refined using the bootstrapping method with 5000 resamples to correct for potential bias and obtain robust estimates of the regression coefficients.

In these adjusted analyses (Table 3), only two sociodemographic variables showed statistically significant associations with GAF: the presence of special educational support (*p* = 0.031) and family socioeconomic classification according to the Brazilian Economic Classification Criteria (ABEP) (*p* = 0.023).

Regarding FSIQ, no statistically significant associations were observed with the sociodemographic or clinical variables included in the models.

In the multivariate CBCL domain models (Table 4), age remained strongly associated with the Withdrawn/Depressed domain, retaining statistical significance after adjustment and bootstrapping (*p* < 0.001). Additionally, male sex was significantly associated with Attention Problems (*p* = 0.017), and socioeconomic status (ABEP) showed an inverse association with Thought Problems (*p* = 0.033). No other CBCL domains reached conventional levels of statistical significance in the adjusted models. Associations with *p*-values between 0.05 and 0.20 are therefore reported as trend-level findings and are presented for exploratory purposes only.

Finally, in the multivariate analysis of Global Adaptive Functioning (GAF), Social Problems showed a trend-level negative association (B = −0.71, *p* = 0.055), consistent with the pattern observed in the univariate model, but not reaching conventional statistical significance (Table 5). Additional trend-level associations were observed for Communication (B = −0.51, *p* = 0.103) and Socialization; however, these findings should be interpreted cautiously and are reported for exploratory purposes only (Table 5).

## 4. Discussion

The present study investigated the sociodemographic, clinical, and intellectual profile (FSIQ) of individuals with 22q11.2 deletion syndrome (22q11.2DS), a well-characterized high-risk neurogenetic copy number variant, with adaptive functioning (GAF) conceptualized as the primary functional outcome, in order to examine its associations with behavioral difficulties and neuropsychiatric risk outcomes.

Regarding intellectual performance, a wide variability in FSIQ scores was observed, ranging from values consistent with intellectual disability (IQ < 70) to those within the average range (IQ > 85). This pattern aligns with previous findings in the literature, which emphasize cognitive heterogeneity as a hallmark feature of individuals with 22q11.2DS [3,14,15]. Such variability is consistent with the notion that the same hemizygous 22q11.2 deletion can give rise to a broad spectrum of neurodevelopmental outcomes, even in the presence of a shared genetic etiology.

In contrast to the cognitive profile most commonly reported in 22q11.2DS—typically centered on borderline intellectual functioning—the present sample showed a predominance of more pronounced cognitive impairment. This pattern of combined intellectual and adaptive impairments is consistent with previous international and Brazilian studies, which have repeatedly documented a high prevalence of mild to moderate intellectual disability in individuals with 22q11.2 deletion syndrome [3,11,13,15,25,34,35].

Part of the intellectual impairment observed in this group may be attributed to the high frequency of medical complications, ranging from perinatal events to neurological and systemic abnormalities. Previous research has demonstrated that secondary medical insults are associated with greater cognitive impairment, reinforcing the cumulative contribution of clinical burden to heightened neuropsychological vulnerability [3,13,36]. From a gene–environment interaction perspective, these complications may act as additional risk factors superimposed on the strong baseline genetic vulnerability conferred by the 22q11.2 deletion.

Another factor that may have contributed to the severity of impairment is that approximately 40% of the children were assessed two years after the COVID-19 pandemic lockdown. This period was marked by disruption of routine medical care, suspension of non-medical interventions—such as speech, occupational, and psychological therapies—and prolonged school closure. Such interruptions in clinical and educational follow-up have been previously described as having a substantial impact on developmental outcomes in individuals with 22q11.2DS [37]. In this context, the pandemic can be viewed as an adverse environmental stressor that potentially amplified the expression of neurodevelopmental risks associated with this CNV.

No significant correlations were found between global adaptive functioning (GAF) scores, as measured by the Vineland Adaptive Behavior Scales, and intellectual performance (FSIQ). This finding contrasts with previous studies in children and adolescents with 22q11.2DS [20,21] but aligns with research in adult samples, where low cognitive and adaptive functioning are considered core clinical features of the syndrome [6,24].

When examining specific adaptive domains, socialization skills appeared relatively preserved compared with other areas, particularly daily living skills related to autonomy and personal care. This pattern corroborates previous reports describing socialization as one of the most resilient aspects of adaptive functioning in individuals with 22q11.2DS, frequently outperforming daily living domains [21].

Multivariate regression models with 5000 bootstrap resamples indicated that the need for special educational support was associated with lower adaptive functioning scores. This finding should not be interpreted as a causal effect of educational support; rather, it suggests that children with more pronounced cognitive and adaptive impairments are more likely to be referred for and to receive specialized pedagogical services, reflecting greater overall clinical severity [11,16,17,18]. Accordingly, this pattern is consistent with reverse causation, whereby higher levels of impairment lead to increased service utilization, rather than educational support shaping adaptive outcomes. These findings underscore the importance of interpreting educational support as a marker of clinical severity when evaluating adaptive functioning in this population [38].

Negative associations were also observed between socioeconomic status (SES) and global adaptive functioning, reaching statistical significance in the adjusted models, indicating that greater adaptive impairment was associated with less favorable socioeconomic conditions. A similar pattern was observed for behavioral difficulties in the CBCL Thought Problems domain, which also reached statistical significance after adjustment, suggesting that lower SES may be linked to increased difficulties in cognitive organization and thought regulation. Socioeconomic disadvantage has been consistently associated with poorer functional outcomes and higher levels of behavioral problems in prior studies [39,40]. Within the context of a genetically determined CNV, these findings underscore how contextual socioeconomic factors may further constrain adaptive and behavioral development, reinforcing the relevance of a multilevel framework that integrates both biological vulnerability and environmental risk in 22q11.2DS.

With respect to behavioral outcomes, age showed a significant association with the CBCL Withdrawn/Depressed domain, indicating that symptoms of social withdrawal—potentially reflecting depressive features—become more pronounced with increasing age [7]. In addition, male sex was significantly associated with higher Attention Problems scores, consistent with previous literature indicating greater attentional difficulties in boys [20]. These age- and sex-related effects suggest that the behavioral expression of the 22q11.2 deletion is dynamic across development and differs between males and females, in line with broader evidence of sex-specific vulnerability in neurodevelopmental disorders.

Finally, social problems showed a trend-level association with global adaptive functioning, suggesting that interpersonal difficulties may be related to greater functional limitations. Associations between social problems and adaptive impairment have been reported in prior studies [40]. Within 22q11.2DS, social difficulties are frequently intertwined with ADHD, anxiety disorders, and psychotic symptoms [40,41]. In the present sample, the observed link between social problems and GAF points to social functioning as a potentially relevant contextual factor in adaptive outcomes, although this finding should be interpreted cautiously given its trend-level nature and the exploratory scope of the analysis.

## 5. Conclusions

This study contributes to the understanding of the cognitive, adaptive, and behavioral profile of children and adolescents with 22q11.2 deletion syndrome (22q11.2DS), a high-risk neurogenetic copy number variant characterized by marked intellectual variability and frequent adaptive impairments. The findings indicate that adaptive functioning represents a relevant component for understanding functional outcomes in this population, being influenced not only by underlying genetic vulnerability but also by contextual factors. Despite limitations, including the small sample size and the retrospective design, this is the first Brazilian study to systematically examine adaptive functioning in a pediatric 22q11.2DS cohort using standardized instruments and robust statistical methods. These results may inform early intervention strategies and support public policies aimed at promoting neurodevelopment, highlighting the need for future longitudinal studies to clarify adaptive trajectories across development.

## Figures and Tables

**Table 1:** Sociodemographic and clinical data of participants (*n* = 34).

Sociodemographic Variables	Min/Max	Mean ± SD	Median
**Age**	01/17	11.5 ± 4.6	13.0
	**N**		**%**
Type of school Public Private No attending school	10 11 03		59 35 09
No literate	06		35
Special educational support	17		50
Caretaker years of schooling Elementary School I Elementary School II High School Higher education Family socioeconomic classification A B C D	01 05 13 14 07 12 15 05		3 18 38 41 21 32 32 14
**Clinical variables**	**N**		**%**
Pregnancy problems	09		27
Neonatal complications	17		50
Health problems *	29		85
Developmental delay	28		82
Maxillofacial abnormalities	11		32
Congenital heart disease	24		71
Neurological problems	07		21
Use of psychotropic medication	11		32
Multidisciplinary follow-up	18		53

Note: = Family socioeconomic status, according to the Brazilian Economic Classification Criteria (ABEP); * Health problems in the first years of life.

**Table 2:** Intellectual, cognitive, adaptive functioning, and behavioral problems profile of participants in standard scores (*n* = 34).

Variáveis	N	Min/Max	Mean ± SD	Median
FSIQ_WISC_WASI	27	45/105	69.3 ± 15.4	68.0
Nonverbal FSIQ_Son_R	2	52/59	55.5 ± 5.0	55.5
Cognitive domain_Bayley	2	75/90	82.5 ± 10.6	82.0
G.A.F._VABS	32	47/125	70.4 ± 16.1	66.5
COM._VABS	32	41/102	66.7 ± 14.3	65.0
D.L.S._VABS	32	29/125	69.9 ± 20.2	66.5
SOC._VABS	32	43/135	73.1 ± 19.9	60.0
MOT._VABS	08	47/125	61.4 ± 16.6	60.0
Anxious_CBCL	28	51/83	63.1 ± 9.3	63.1
Withdrawn__CBCL	28	50/100	**67.1 ± 14.1**	67.1
Simatic__CBCL	28	32/82	60.1 ± 10.6	60.1
Rule-Breaking.__CBCL	28	50/71	57.9 ± 6.8	57.9
Agressive Beha.__CBCL	28	50/76	59.4 ± 8.1	59.4
Social Probl.__CBCL	28	54/88	**66.6 ± 9.5**	66.6
Thought Probl.__CBCL	28	50/78	63.6 ± 8.3	63.6
Attention Probl.__CBCL	28	52/93	**67.9 ± 10.5**	67.5

Note: Results expressed as standard scores < 70 = deficit; 70–79 = borderline; 80–89 = low average; 90–109 = average; 110–119 = high average; 120–129 = superior; >130 = very superior; FSIQ = Full-Scale IQ; Nonverbal FSIQ = Nonverbal Intelligence Quotient, SON-R Test; GAF = Global Adaptive Functioning; COM = Communication; DLS = Daily Living Skills; SOC = Socialization; MOT = Motor Skills; Somatic = Somatic Complaints; Rule-Breaking = Rule-Breaking Behavior; Aggressive Beha.= Aggressive Behavior; Social Probl. = Social Problems; Thought Probl. = Thought Problems; Attention Probl. = Attention Problems; CBCL T-score > 65; Min = Minimum; Max = Maximum; SD = Standard Deviation.

**Table 3:** Multivariate regression models with bootstrapping (5000 samples) for FSIQ and GAF.

Variables	B	ES	*p*	CI 95%
**FSIQ ** ^a^				
**Sociodemographic variables**				
Type of school	10.96	9.61	0.271	(−10.28/27.65)
Literate	9.48	6.14	0.120	(−4.59/20.11)
Family social class	−0.53	4.58	0.908	(−9.21/8.59)
**Clinical variables**				
Developmental delay	−13.06	13.36	0.479	(−33.87/7.59)
Neurological problems	−12.94	7.49	0.088	(−25.68/−0.20)
**GAF ** ^b^				
**Sociodemografic variables**				
Special educational support	−12.54	5.11	**0.031**	(−23.05/−3.00)
Family social class	−6.83	2.46	**0.023**	(−11.66/−1.79)
**Clinical variables**				
Health problems	−15.12	12.63	0.256	(−42.17/7.00)
Developmental delay	−9.65	5.88	0.114	(−20.57/2.64)
Use of psychotropic medication	−4.12	4.26	0.353	(−13.16/4.15)

Note: SE = Standard Error; CI = Confidence Interval; Family social class = Brazilian Association of Research Companies ABEP; FSIQ = Full-Scale IQ; GAF = Global Adaptive Functioning. Statistically significant values are highlighted in bold. ^a^ Sociodemographic data: R^2^ = 0.24; R^2^adj = 0.13, F(3,22) = 2.27, *p* = 0.109; Clinical data: R^2^ = 0.20; R^2^adj = 0.10, F(2,17) = 2.10, *p* = 0.153. ^b^ Sociodemographic data: R^2^ = 0.25; R^2^adj = 0.15, F(3,22) = 2.47, *p* = 0.089; Clinical data: R^2^ = 0.22; R^2^adj = 0.14, F(3,27) = 2.56, *p* = 0.076.

**Table 4:** Multivariate regression models with bootstrapping (5000 samples) for CBCL.

Variables	B	SE	*p*	CI 95%
**Anxious ** ^a^				
**Sociodemographic variables**				
Gender	4.81	3.77	0.210	(−3.06/12.09)
Family social class	−2.90	1.78	0.125	(−6.45/0.48)
**Clinical variables**				
Inherited deletion	−4.00	3.03	0.166	(−8.50/2.75)
Neurological problems	−4.04	3.75	0.299	(−11.77/2.51)
**Withdrawn ** ^b^				
**Sociodemographic variables**				
Age	1.95	0.53	**<0.001**	(0.95/2.99)
**Clinical variables**				
Congenital heart disease	−13.16	6.03	0.050	(−25.09/−0.97)
Use of psychotropic medication	−5.76	5.46	0.302	(−16.11/5.28)
**Somatic ** ^c^				
**Sociodemographic variables**				
Age	0.49	0.75	0.507	(−0.92/2.12)
Literate	10.89	6.51	0.090	(−1.42/23.50)
**Clinical variables**				
Inherited deletion	−18.00	9.81	0.083	(−36.85/1.19)
Pregnancy problems	−2.92	4.12	0.506	(−11.77/4.09)
Neurological problems	−0.45	4.20	0.926	(−8.30/8.00)
**Social Problems ** ^d^				
**Clinical variables**				
Inherited deletion	9.98	5.99	0.076	(1.00/23.00)
Congenital heart disease	−7.69	4.72	0.119	(−16.35/2.30)
**Thought Problems ** ^e^				
**Sociodemographic variables**				
Gender	5.13	2.73	0.071	(0.17/10.86)
Type of school	−0.28	3.94	0.946	(−8.63/7.14)
Family social class	−4.17	1.87	**0.033**	(−7.61/−0.14)
**Clinical variables**				
Health problems	4.44	3.23	0.165	(−2.53/10.38)
**Attention Problems ** ^f^				
**Sociodemographic variables**				
Gender	10.75	3.74	**0.017**	(3.64/18.14)
**Clinical variables**				
Congenital heart disease	−7.77	6.25	0.237	(−20.62/4.08)
Neurological problems	−9.90	5.68	0.096	(−20.98/1.56)

Note: SE = Standard Error; CI = Confidence Interval; Family social class = Brazilian Association of Research Companies ABEP; Statistically significant values are highlighted in bold. ^a^ Sociodemographic data: R^2^ = 0.32; R^2^adj = 0.23, F(3,22) = 3.46, *p* = 0.034; Clinical data: R^2^ = 0.11; R^2^adj = 0.01, F(2,19) = 1.12, *p* = 0.347. ^b^ Sociodemographic data: R^2^ = 0.18; R^2^adj = 0.14, F(1,26) = 5.54, *p* = 0.026; Clinical data: R^2^ = 0.24; R^2^adj = 0.17, F(2,22) = 3.48, *p* = 0.049. ^c^ Sociodemographic data: R^2^ = 0.20; R^2^adj = 0.13, F(2,23) = 2.93, *p* = 0.073; Clinical data: R^2^ = 0.38; R^2^adj = 0.26, F(3,15) = 3.06, *p* = 0.061. ^d^ Sociodemographic data: R^2^ = 0.05; R^2^adj = 0.01, F(1,26) = 1.39, *p* = 0.249; Clinical data: R^2^ = 0.24; R^2^adj = 0.17, F(2,22) = 3.52, *p* = 0.047. ^e^ Sociodemographic data: R^2^ = 0.42; R^2^adj = 0.35, F(3,24) = 5.84, *p* = 0.004; Clinical data: R^2^ = 0.20; R^2^adj = 0.13, F(2,25) = 3.04, *p* = 0.066. ^f^ Sociodemographic data: R^2^ = 0.27; R^2^adj = 0.24, F(1,26) = 9.38, *p* = 0.005; Clinical data: R^2^ = 0.22; R^2^adj = 0.12, F(2,16) = 2.20, *p* = 0.143.

**Table 5:** Multivariate regression models with bootstrapping (5000 samples) for psychometric outcomes.

Variables	B	SE	*p*	CI 95%
**GAF ** ^a^				
Social Problems	−0.71	0.35	**0.055**	(−1.45/−0.09)
**Adaptive Functioning—Communication ** ^b^				
Social Problems	−0.51	0.30	0.103	(−1.04/0.15)
**Adaptive Functioning—Socialization ** ^c^				
Social Problems	−0.73	0.64	0.278	(−2.09/0.41)
**Attention Problems**	−0.08	0.55	0.870	(−0.81/1.37)

Note: SE = Standard Error; CI = Confidence Interval; Statistically significant values are highlighted in bold. ^a^ R^2^ = 0.16; R^2^_adj_ = 0.13; F(1,24) = 4.61; *p* = 0.042; ^b^ R^2^ = 0.11; R^2^_adj_ = 0.08; F(1,24) = 3.02; *p* = 0.095; ^c^ R^2^ = 0.13; R^2^_adj_ = 0.05; F(1,23) = 1.69; *p* = 0.207.

## Data Availability

The data supporting this study’s findings are available from the corresponding author upon reasonable request.

## Appendix A

### Appendix A.1. Univariate Linear Regression Analyses Between Sociodemographic, Clinical Variables and FSIQ—Only p < 0.200 (n = 34)

**Table:**

Variables	B	SE	*p*	CI 95%
**FSIQ**				
**Sociodemographic variables**				
Age	1.54	1.01	0.142	(−0.54/3.63)
Type of school	14.68	5.94	**0.021**	(2.46/26.90)
Literate	14.13	7.99	0.089	(−2.35/30.62)
Caretaker years of schooling	5.71	3.39	0.103	(−1.25/12.67)
Family social class	−5.66	2.83	0.056	(−11.46/0.17)
**Clinical variables**				
Developmental delay	−13.56	7.34	0.077	(−28.70/1.58)
Neurological problems	−14.06	7.89	0.091	(−30.56/2.45)
**GAF**				
**Sociodemografic variables**				
Special educational support	−9.43	5.52	0.098	(−20.70/1.88)
Family social class	−5.29	2.85	**0.073**	(−11.10/0.52)
**Clinical variables**				
Health problems	−17.13	7.30	**0.026**	(−32.05/−2.22)
Developmental delay	−10.95	7.84	0.173	(−26.99/5.10)
Use of psychotropic medication	−8.36	6.03	0.176	(−20.68/3.97)

Note: SE = Standard Error; CI = Confidence Interval; Family social class = Brazilian Association of Research Companies ABEP; FSIQ = Full-Scale IQ; GAF = Global Adaptive Functioning; Statistically significant values are highlighted in bold.

### Appendix A.2. Univariate Linear Regression Analyses Between Sociodemographic, Clinical Variables and CBCL—Only p < 0.200 (n = 34)

**Table:**

Variant	B	SE	*p*	CI 95%
**Anxious**				
**Sociodemographic variables**				
Gender	6.38	3.41	0.073	(−0.63/13.38)
Family social class	−3.57	1.70	**0.046**	(−7.06/−0.07)
**Clinical variables**				
Inherited deletion	−9.46	4.78	0.059	(−19.29/0.38)
Neurological problems	−6.04	4.52	0.171	(−14.91/2.83)
**Withdrawn**				
**Sociodemographic variables**				
Age	1.95	0.83	**0.026**	(0.25/3.66)
**Clinical variables**				
Congenital heart disease	−14.57	5.94	**0.022**	(−26.86/−2.29)
Use of psychotropic medication	−7.83	5.75	0.185	(−19.67/4.00)
**Somatic**				
**Sociodemographic variables**				
Age	0.96	0.65	0.154	(−0.38/2.30)
Literate	11.66	4.90	**0.026**	(1.54/21.77)
**Clinical variables**				
Inherited deletion	−11.79	5.32	**0.035**	(−22.71/−0.87)
Pregnancy problems	−7.52	4.45	0.104	(−16.72/1.68)
Neonatal complications	−5.62	4.07	0.181	(−14.02/2.79)
Neurological problems	−9.40	4.28	**0.040**	(−18.32/−0.47)
**Social Problems **				
**Clinical variables**				
Inherited deletion	8.28	5.68	0.157	(−3.40/19.96)
Congenital heart disease	−8.56	4.04	**0.045**	(−16.93/−0.20)
**Thought Problems **				
**Sociodemographic variables**				
Gender	7.40	2.88	**0.016**	(1.47/13.32)
Type of school	5.28	3.17	0.108	(−1.23/11.79)
Family social class	−4.79	1.34	**0.001**	(−7.54/−2.03)
**Clinical variables**				
Health problems	5.67	4.42	0.212	(−3.43/14.76)
**Attention Problems**				
**Sociodemographic variables**				
Gender	10.75	3.51	**0.005**	(3.53/17.97)
**Clinical variables**				
Congenital heart disease	−7.64	4.69	0.117	(−17.33/2.06)
Neurological problems	−9.50	5.91	0.093	(−20.75/1.75)

Note: SE = Standard Error; CI = Confidence Interval; Family social class = Brazilian Association of Research Companies ABEP; FSIQ = Full-Scale IQ; GAF = Global Adaptive Functioning; Statistically significant values are highlighted in bold.

### Appendix A.3. Univariate Linear Regression Analyses Among Psychometric Outcomes—Only p < 0.200 (n = 34)

**Table:**

Variables	B	SE	*p*	CI 95%
**GAF**				
Social Problems	−0.71	0.33	**0.042**	(−1.40/−0.03)
**Adaptive Functioning—Communication**				
Social Problems	−0.51	0.29	0.095	(−1.19/0.10)
**Adaptive Functioning—Socialization**				
Social Problems	−0.79	0.42	0.074	(−1.66/0.08)
**Attention Problems**	−0.53	0.41	0.199	(−1.37/0.30)

Note: SE = Standard Error; CI = Confidence Interval; Family social class = Brazilian Association of Research Companies ABEP; FSIQ = Full-Scale IQ; GAF = Global Adaptive Functioning; Statistically significant values are highlighted in bold.

## References

[1] Babcock M., Pavlicek A., Spiteri E., Kashork C.D., Ioshikhes I., Shaffer L.G., Jurka J., Morrow B.E. (2003). Shuffling of genes within low-copy repeats on 22q11 (LCR22) by Alu-mediated recombination events during evolution. Genome Res..

[2] Cirillo A., Lioncino M., Maratea A., Passariello A., Fusco A., Fratta F., Monda E., Caiazza M., Signore G., Esposito A. (2022). Clinical manifestations of 22q11.2 deletion syndrome. Heart Fail. Clin..

[3] McDonald-McGinn D.M., Sullivan K.E., Marino B., Philip N., Swillen A., Vorstman J.A.S. (2015). 22q11.2 deletion syndrome. Nat. Rev. Dis. Primers.

[4] Shprintzen R.J., Higgins A.M., Antshel K., Fremont W., Roizen N., Kates W. (2005). Velo-cardio-facial syndrome. Curr. Opin. Pediatr..

[5] Radoeva P.D., Fremont W., Antshel K.M., Kates W.R. (2017). Longitudinal study of premorbid adjustment in 22q11.2 deletion syndrome and association with psychosis. Dev. Psychopathol..

[6] Schneider M., Debbané M., Bassett A.S., Chow E.W.C., Fung W.L.A., van den Bree M., Owen M., Murphy K.C., Niarchou M., Kates W.R. (2014). Psychiatric disorders from childhood to adulthood in 22q11.2 deletion syndrome. Am. J. Psychiatry.

[7] Fabbro A., Rizzi E., Schneider M., Debbané M., Eliez S. (2012). Depression and anxiety disorders in children and adolescents with velo-cardio-facial syndrome. Eur. Child Adolesc. Psychiatry.

[8] Gothelf D., Schneider M., Green T., Debbané M., Frisch A., Glaser B., Zilkha H., Schaer M., Weizman A., Eliez S. (2013). Risk factors and the evolution of psychosis in 22q11.2 deletion syndrome. J. Am. Acad. Child Adolesc. Psychiatry.

[9] Fiksinski A.M., Schneider M., Murphy C.M., Armando M., Vicari S., Canyelles J.M., Gothelf D., Eliez S., Breetvelt E.J., Arango C. (2018). Understanding the pediatric psychiatric phenotype of 22q11.2 deletion syndrome. Am. J. Med. Genet. A.

[10] Swillen A., Moss E., Duijff S. (2018). Neurodevelopmental outcome in 22q11.2 deletion syndrome and management. Am. J. Med. Genet. A.

[11] De Smedt B., Devriendt K., Fryns J.P., Vogels A., Gewillig M., Swillen A. (2007). Intellectual abilities in a large sample of children with velo-cardio-facial syndrome: An update. J. Intellect. Disabil. Res..

[12] Jacobson C., Shearer J., Habel A., Kane F., Tsakanikos E., Kravariti E. (2010). Core neuropsychological characteristics of children and adolescents with 22q11.2 deletion. J. Intellect. Disabil. Res..

[13] Swillen A., McDonald-McGinn D. (2015). Developmental trajectories in 22q11.2 deletion syndrome. Am. J. Med. Genet. C Semin. Med. Genet..

[14] Pimenta L.S.E., Mello C.B., Soares D.C.Q., Dantas A.G., Melaragno M.I., Kulikowski L.D., Kim C.A. (2023). Intellectual performance profile of Brazilian children and adolescents with 22q11.2 deletion syndrome. Estud. Psicol..

[15] Zhao Y., Guo T., Fiksinski A., Breetvelt E., McDonald-McGinn D.M., Crowley T.B., Diacou A., Schneider M., Eliez S., Swillen A. (2018). Variance of IQ is partially dependent on deletion type among 22q11.2 deletion syndrome subjects. Am. J. Med. Genet. A.

[16] Antshel K.M., Shprintzen R., Fremont W., Higgins A.M., Faraone S.V., Kates W.R. (2010). Cognitive and psychiatric predictors of psychosis in velocardiofacial syndrome. J. Am. Acad. Child Adolesc. Psychiatry.

[17] Moss E.M., Batshaw M.L., Solot C.B., Gerdes M., McDonald-McGinn D.M., Driscoll D.A., Emanuel B.S., Zackai E.H., Wang P.P. (1999). Psychoeducational profile of the 22q11.2 microdeletion. J. Pediatr..

[18] Niklasson L., Rasmussen P., Óskarsdóttir S., Gillberg C. (2009). Autism, ADHD, intellectual disability and behavior problems in 22q11 deletion syndrome. Res. Dev. Disabil..

[19] World Health Organization (WHO) (2008). International Classification of Functioning, Disability and Health (ICF).

[20] Antshel K.M., Abdulsabur N., Roizen N., Fremont W., Kates W.R. (2005). Sex differences in cognitive functioning in velocardiofacial syndrome. Dev. Neuropsychol..

[21] Dewulf D., Noens I., Swillen A. (2013). Adaptive skills, cognitive functioning and behavioural problems in adolescents with 22q11.2 deletion syndrome. Tijdschr. Psychiatr..

[22] Wallin L., Gillberg C., Knutsson J., Fernell E., Gillberg I.C., Billstedt E. (2025). Cognitive, visuomotor and adaptive functioning in 22q11.2 deletion syndrome: A longitudinal study. Brain Behav..

[23] Angkustsiri K., Leckliter I., Tartaglia N., Beaton E.A., Enriquez J., Simon T.J. (2012). Anxiety, intelligence and adaptive functioning in children with chromosome 22q11.2 deletion syndrome. J. Dev. Behav. Pediatr..

[24] Vingerhoets C., Ruiz-Fernandez J., Scheibler E., Vergaelen E., Volbragt N., Soons N., Serrarens C., Vogels A., Boot E., van Amelsvoort T. (2024). Cognitive, adaptive and daily life functioning in adults with 22q11.2 deletion syndrome. BJPsych Open.

[25] ABEP (2008). Critério de Classificação Econômica Brasil.

[26] Bayley N. (2017). Bayley Scales of Infant and Toddler Development.

[27] Laros J.A., Tellegen P.J., Jesus G.R., Karino C.A. (2015). SON-R 2½–7[a]: Nonverbal Intelligence Test—Brazilian Standardization.

[28] Wechsler D. (2014). Wechsler Abbreviated Scale of Intelligence (WASI): Manual.

[29] Wechsler D. (2013). Wechsler Intelligence Scale for Children—Fourth Edition (WISC-IV): Administration and Scoring Manual.

[30] Sparrow S.S., Cicchetti D.V., Balla D.A. (2016). Vineland Adaptive Behavior Scales.

[31] Sparrow S.S., Cicchetti D.V., Saulnier C.A. (2019). Vineland Adaptive Behavior Scales.

[32] Achenbach T.M., Ruffle T.M. (2000). The Child Behavior Checklist and related forms. Pediatr. Rev..

[33] Hosmer D.W., Lemeshow S., Sturdivant R.X. (2013). Applied Logistic Regression.

[34] Pimenta L.S.E. (2019). Neurocognitive and Behavioral Phenotype of 22q11.2 Deletion Syndrome. Ph.D. Thesis.

[35] Pimenta L.S.E., Mello C.B., Benedetto L.M.D., Soares D.C.Q., Kulikowski L.D., Dantas A.G., Melaragno M.I., Kim C.A. (2024). Neuropsychological profile of Brazilian patients with 22q11.2 deletion syndrome. Genes.

[36] Cheung E.N.M., George S.R., Andrade D.M., Chow E.W.C., Silversides C.K., Bassett A.S. (2014). Neonatal hypocalcemia and neurodevelopmental outcome in 22q11.2 deletion syndrome. Genet. Med..

[37] White L.K., Crowley T.B., Finucane B., Garcia-Minaur S., Repetto G.M., van den Bree M., Fischer M., Jacquemont S., Barzilay R., Maillard A.M. (2022). Impact of the COVID-19 pandemic in individuals with 22q11.2 copy number variants. J. Intellect. Disabil. Res..

[38] Bassett A.S., McDonald-McGinn D.M., Devriendt K., Digilio M.C., Goldenberg P., Habel A., Marino B., Oskarsdottir S., Philip N., Sullivan K. (2011). Practical guidelines for managing patients with 22q11.2 deletion syndrome. J. Pediatr..

[39] Snihirova Y., Linden D.E.J., van Amelsvoort T., van der Meer D. (2022). Environmental influences on mental health in 22q11.2 deletion syndrome. Genes.

[40] Shashi V., Veerapandiyan A., Schoch K., Kwapil T., Keshavan M., Ip E., Hooper S. (2012). Social skills and psychopathology in children with chromosome 22q11.2 deletion syndrome. J. Intellect. Disabil. Res..

[41] Debbané M., Glaser B., David M.K., Feinstein C., Eliez S. (2006). Psychotic symptoms in youth with 22q11.2 deletion syndrome. Schizophr. Res..

